# Fuzzy Neural Network-Based Interacting Multiple Model for Multi-Node Target Tracking Algorithm

**DOI:** 10.3390/s16111823

**Published:** 2016-11-01

**Authors:** Baoliang Sun, Chunlan Jiang, Ming Li

**Affiliations:** State Key Laboratory of Explosion Science and Technology, Beijing Institute of Technology, Beijing 100081, China; jiangchunwh@bit.edu.cn (C.J.); iseagle@ bit.edu.cn (M.L.)

**Keywords:** wireless sensor network, multi-sensing data fusion, interacting multiple model, fuzzy neural network, target tracking

## Abstract

An interacting multiple model for multi-node target tracking algorithm was proposed based on a fuzzy neural network (FNN) to solve the multi-node target tracking problem of wireless sensor networks (WSNs). Measured error variance was adaptively adjusted during the multiple model interacting output stage using the difference between the theoretical and estimated values of the measured error covariance matrix. The FNN fusion system was established during multi-node fusion to integrate with the target state estimated data from different nodes and consequently obtain network target state estimation. The feasibility of the algorithm was verified based on a network of nine detection nodes. Experimental results indicated that the proposed algorithm could trace the maneuvering target effectively under sensor failure and unknown system measurement errors. The proposed algorithm exhibited great practicability in the multi-node target tracking of WSNs.

## 1. Introduction

Wireless sensor networks (WSNs) are developing toward positive intelligence, a large scale, modularization, and integration with the advancement of sensors, electronic information, and integrated technology [1,2]. Given its technological superiority, including self-organization, fast deployment, high fault tolerance, and strong elusion, WSNs are extremely suitable for various applications, such as battlefield target location [3], intelligent transportation system [4], and ocean exploration [5].

The maneuvering target tracking technology is one of the current research hot spots in WSNs [6,7,8,9]. This technology uses multi-sensor data to conduct state estimations on targets. Numerous maneuvering target tracking algorithms are presently available; among those methods, the interacting multiple model (IMM) algorithm has been extensively applied, considering algorithm performance, complexity, storage, and engineering applied demands [10,11]. This method uses interacting integration among multiple maneuvering models to describe target motion state. The transition between models is controlled by a Markov chain [12,13]. The weakness of IMM is that it requires knowledge of the statistical property of noise signals, unknown in most situations. Several methods have been introduced recently to solve target tracking problems, and many scholars have introduced fuzzy technology into the IMM algorithm to estimate model parameters in real time. For example, a fuzzy logic-based adaptive Kalman filter was proposed in [14]; its main concept involves combining a fuzzy inference machine with a Kalman filter and realizing adaptive Kalman filtering by utilizing the excellent performance of fuzzy logical processing with inaccurate data and simple operations. In [15], a sensor fusion algorithm was proposed, which introduced a dynamic noise covariance matrix into the IMM. The proposed filter exhibits superior accuracy compared with a Kalman filter when a vehicle abruptly changes its trajectory. Several algorithms that combine fuzzy technology with the IMM algorithm have been introduced in the literature [16,17,18], and such a combination has provided good state estimation results. A fuzzy-logic-based interacting multiple model (FIMM) algorithm for tracking a maneuvering target was proposed in [16]. A fuzzy system enhanced by a genetic algorithm is applied as a universal approximator to compute the variance of the overall process noise, which is time-varying because of the unknown target acceleration. The FIMM algorithm does not require prior information on the statistical properties of the target maneuver but the statistical characteristics of measurement errors. In [17], a fuzzy Kalman filter was proposed to combat the model-set adaptation problem and was found to be able to adaptively control the noise covariance such that the IMM algorithm can use an accurate model set to improve its performance. In [18], fuzzy logic was incorporated in a conventional IMM-PDA (probabilistic data association) method, which adopted the prediction error and change of the prediction error as fuzzy inputs. The feasibility of this proposed algorithm was examined by simulation.

The Takagi–Sugeno fuzzy neural network (T–S FNN) model [19,20] is a fuzzy reasoning model proposed by Takagi and Sugeno that is widely applied in time-series prediction and parameter estimation [21,22]. The T–S FNN fuzzy inference machine can be realized using a combined neural network. An agile ENFS, termed PANFIS, was explored in [23]. PANFIS can commence its learning process from scratch with an empty rule base, and the fuzzy rules can be stitched up and expelled through the statistical contributions of fuzzy rules and injected datum afterward [23]. In [24], the author presented an architecture and learning procedure underlying an adaptive-network-based fuzzy inference system (ANFIS), which could construct input–output mapping based on both human knowledge and stipulated input–output data pairs. The proposed architecture of ANFIS can refine fuzzy if–then rules obtained from human experts. If human expertise is unavailable, then the proposed method can generate a set of fuzzy if–then rules by intuitively setting up reasonable initial membership functions and starting the learning process.

Multi-node or multi-sensor data fusion is seldom introduced in these studies. A FNN-based IMM (FNN–IMM) filter is proposed in this work. Assuming each sensor node in a WSN to correspond to an FNN–IMM filter, several FNN–IMM filters were operated in parallel. The output results of all FNN–IMM filters were inputted into the fusion center during the multi-sensor data fusion stage to conduct data fusion. Finally, a network state estimation of the targets was obtained. The algorithm proposed in this study did not require the assumption of the statistical characteristics of measurement errors and exhibited good tracking performance in the network when some nodes outputted error data.

This work is divided into five parts, including this section. The FNN–IMM algorithm of a single node is described in detail in Section 2. We discuss the multi-node target tracking and propose the multi-node tracking data fusion algorithm in Section 3. The capabilities of the proposed method are discussed and validated through simulation in Section 4. Finally, conclusions are drawn in Section 5.

## 2. The FNN–IMM Algorithm of Single Node

A WSN can be divided into multiple structures based on networking modes: star configuration, symmetrical structure, dendritical structure, and mixed structure. The following hypotheses are proposed based on network power consumption and tactic utilization.

1. N detection nodes with the same type are present in a WSN, and the detection nodes only have one detection mode.
2. Data measured by node detectors are 2D position coordinates of the invasion targets.

When a single node is considered, the measuring model is shown as follows:

(1) $$Z(k)=H_{j}X(k)+V_{j}(k)\;j=1,\cdots ,m$$

The target state transition equation is given as follows:

(2) $$X(k+1)=F_{j}X(k)+G_{j}W_{j}(k),\;j=1,\cdots ,m$$

where *m* is the model number in the IMM algorithm, **X**(*k*) is the state vector, **F***_j_* is the system transition matrix, **G***_j_* is the noise disturbance matrix, and **H** is the measurement matrix. **W***_j_* and **V***_j_* are the white noise sequences, and their means are 0; their covariance matrices are **Q***_j_* and **R**, respectively. The transition between models is controlled using a Markov chain, and the transition matrix of the Markov chain is shown as follows:

(3) $$\Pi =\left[\begin{array}{ccc} \pi_{11} & \cdots & \pi_{1m}\\ \vdots & \ddots & \vdots \\ \pi_{m1} & \cdots & \pi_{mm} \end{array}\right]$$

**Figure 1:**
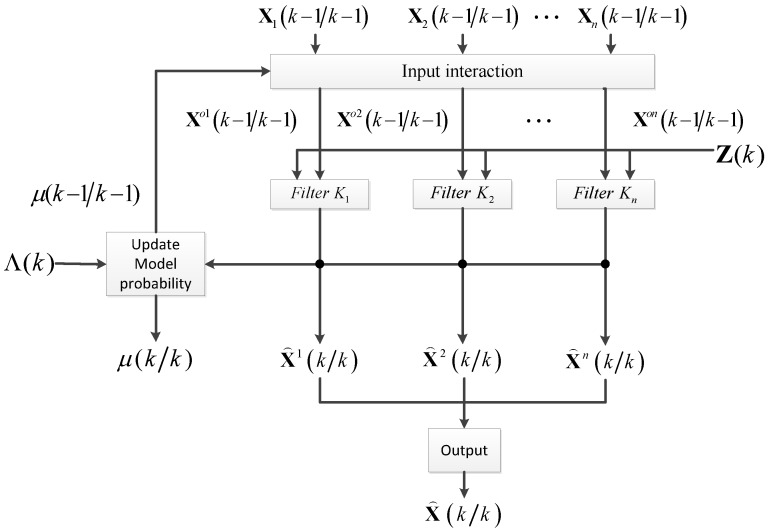
Interacting multiple model (IMM) algorithm principle frame.

Figure 1 illustrates the principle of the IMM algorithm.

The process of the IMM algorithm can be described using Equations (4)–(14).

a. Input interaction:

(4) $${\hat{X}}^{oj}(k-1/k-1)=\sum_{i=1}^{m}{\hat{X}}^{i}(k-1/k-1)\mu_{ij}(k-1/k-1),\;j=1,\cdots ,m$$

and

(5) $$\begin{array}{l} P^{oj}(k-1/k-1)=\sum_{i=1}^{m}\mu_{ij}(k-1/k-1)\{P^{i}(k-1/k-1)\\ +[{\hat{X}}^{i}(k-1/k-1)-{\hat{X}}^{oi}(k-1/k-1)][{\hat{X}}^{i}(k-1/k-1)-{\hat{X}}^{oi}(k-1/k-1){]}^{T}\} \end{array}$$

where

(6) $$\mu_{ij}(k-1/k-1)=\pi_{ij}\mu_{ij}(k-1)/\sum_{i=1}^{m}\pi_{ij}\mu_{ij}(k-1)$$

b. For the *j*-th mode:

Prediction:

(7) $${\hat{X}}^{j}(k/k-1)=F_{j}{\hat{X}}^{oj}(k-1/k-1)$$

Error covariance prediction:

(8) $$P^{j}(k/k-1)=F_{j}P^{oj}(k-1/k-1)F_{j}^{T}+G_{j}Q_{j}G_{j}^{T}$$

Kalman gain:

(9) $$K_{j}(k)=P^{j}(k/k-1){H_{j}}^{T}{\left[H_{j}P^{j}(k/k-1){H_{j}}^{T}+R_{j}\right]}^{-1}$$

Filter:

(10) $${\hat{X}}^{j}(k/k)={\hat{X}}^{j}(k/k-1)K_{j}(k)[Z(k)-H_{j}{\hat{X}}^{j}(k/k-1)$$

Error covariance of filter:

(11) $$P^{j}(k/k)=[I-K_{j}(k)]P^{j}(k/k-1)$$

Model probability updating:

(12) $$\mu_{j}(k)=\mu_{j}(k/k-1)\Lambda_{j}(k)/\sum_{i=1}^{m}\mu_{i}(k-1)\Lambda_{i}(k)$$

where **Λ***_j_*(*k*) is the likelihood function of observational **Z**(*k*), and

(13) $$\Lambda_{i}\left(k\right)=N\left(\nu_{i}\left(k\right);0,S_{j}\left(k\right)\right)=\frac{1}{{\left(2\pi \right)}^{1/2}{\left|S_{j}\left(k\right)\right|}^{1/2}}\exp\left\{-\frac{1}{2}\nu_{j}^{T}\left(k\right)S_{j}^{-1}\left(k\right)\nu_{i}\left(k\right)\right\},$$

where

(14) $$\upsilon_{j}(k)=Z(k)-H(k){\hat{X}}^{j}(k/k-1)$$

and

(15) $$S_{j}(k)=H(k)P^{j}(k/k-1)H^{T}(k)+R$$

c. Interacting output:

(16) $$\hat{X}(k/k)=\sum_{j=1}^{m}{\hat{X}}^{j}(k/k)\mu_{j}(k)$$

(17) $$P(k/k)=\sum_{j=1}^{m}\mu_{j}(k)\{P^{j}(k/k)+[{\hat{X}}^{j}(k/k)-\hat{X}(k/k)]{\left[{\hat{X}}^{j}(k/k)-\hat{X}(k/k)\right]}^{T}\}$$

The algorithm proposed in this study discusses system residual ε(k) of the models during the interacting output stage.

(18) $$\varepsilon (k)=Z(k)-H(k)\hat{X}(k/k)$$

According to [25,26], the theoretical value of the covariance matrix of the system residual is:

(19) $$T(k)=H(k)P(k/k)H^{T}(k)+R$$

However, **T**(*k*) can be estimated but cannot be known in advance because **R** is unknown. We initialized **R** with a random value **R***_r_*. During the iteration progress, the algorithm adaptively adjusted **R***_r_* to the real value **R**. To achieve this self-adaptive progress, we set up a statistical value of the system residual as follows:

(20) $$E(k)=\frac{1}{W}\sum_{i=i_{0}}^{k}\varepsilon (i)\varepsilon {\left(i\right)}^{T},j=1,2,\cdots ,m$$

where *i*_0_ = *k* − *W* + 1, and *W* is the time window length. The proposed algorithm applies the latest *W* datum to estimate the covariance matrix of the system residual. Given that the properties of target maneuvering are unknown, the performance of the IMM algorithm decreases when the target changes its trajectory abruptly, which makes ε(k), **T***(k)*, and **E***(k)* difficult to estimate. A long time window covers much motion progress of the target, which leads to many maneuvering errors. By contrast, a short time window gains minimal statistical data, which cause significant error in **E***(k)*. Therefore, the value of time window *W* is selected according to the sampling rate and maneuvering state of the target. Section 3 presents the simulation part. The operational value of *W* after trial and error is the most ideal when *W* = 15.

Let

(21) EoR=T(k)−E(k)

Equation (19) and (21) indicate that when the covariance matrix **R** of the measuring error increases or decreases, the corresponding elements in the **EoR** matrix also increase or decrease. **EoR** detection can be used to adjust **R** and reduce the difference between **T**(k) and **E**(k). Only the elements on the principal diagonal of **EoR** can be detected because the elements of matrix **R** are 0, except for those elements on the principal diagonal that are not completely 0. 

The principles of adaptive adjustment are presented as follows:

1. diag(**EoR**) is used to express the principal diagonal elements of **EoR**. If diag(**EoR**) ≈ 0, then **R** remains unchanged.
2. If diag(**EoR**) > 0, then the corresponding elements of **R** decrease.
3. If diag(**EoR**) < 0, then the corresponding elements of **R** increase.

Therefore, **R** can be adjusted as follows:

(22) $$R(i,i)=R(i,i)+\Delta R_{i}$$

**Figure 2:**
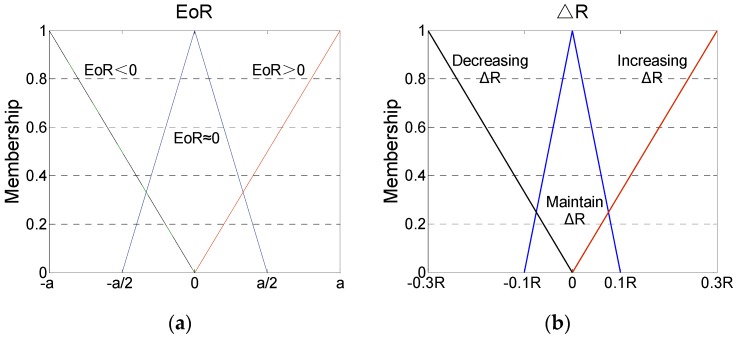
Membership functions of (**a**) **EoR** and (**b**) Δ**R**.

The membership functions of **EoR** and Δ**R** are established as shown in Figure 2. 

In this figure,

(23) $$a=\frac{1}{r}\sum_{i=1}^{r}\left|EoR(i,i)\right|$$

where *r* is the dimension of the matrix **EoR**.

The maximum–minimum principles are utilized in the defuzzification progress:

(24) $$\Delta R\left(i,i\right)=\frac{\mu_{EoR1}f_{R1}(\mu_{EoR1})+\mu_{EoR2}f_{R2}(\mu_{EoR2})}{\mu_{EoR1}+\mu_{EoR2}}$$

where $f_{Ri}(•)$ (*i* = 1, 2, 3) represents the inverse function of △**R** under the corresponding fuzzy rules; $\mu_{EoRi}$(*i* = 1, 2) represents the membership degree of **EoR**.

In a real environment, **R** varies on a per-environment basis. In this study, we detect the change of **R** by examining **EoR**. The algorithm adjusts R adaptively during iteration progress to decrease the tracking error of target. However, the relationship between **EoR** and Δ**R** is unclear because the measure environment is unknown. The FNN is introduced to learn this relationship and rebuild the membership functions of **EoR** and Δ**R**. During the iteration progress, we feed the **EoR** and Δ**R** obtained in each iteration into the FNN and use the output to adjust **R** online.

**Figure 3:**
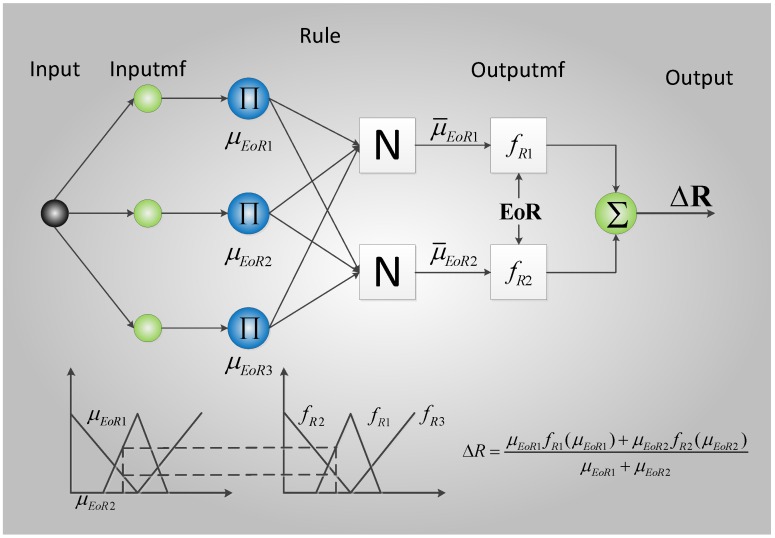
Structure of the single-node fuzzy neural network (FNN) inference machine.

The T–S FNN model is used in this study to construct the fuzzy inference machine between **EOR** and Δ**R** and adaptively deduce the relationship between learning **EOR** and **R**. The FNN inference machine between **EoR** and **R** is established using MATLAB according to [24], and its structure is type 3 of [24], as shown in Figure 3. 

## 3. Multi-Node Target Tracking Data Fusion Algorithm

**Figure 4:**
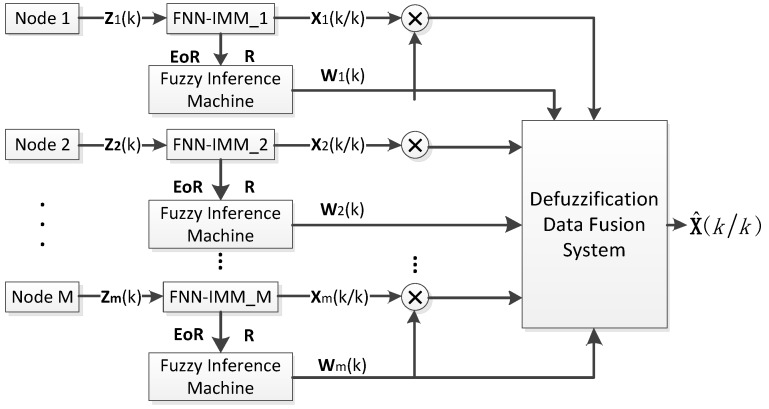
Principle frame graph of FNN fusion system (FNNFS).

During the multi-node data fusion stage, a large amount of sensor data is sent to the infusion center for infusion in WSNs; thus, wrong data inevitably appear. The data infusion algorithm must possess high fault tolerance and efficiency. The FNN fusion system (FNNFS) proposed in this study estimates the working status of nodes with **EoR** and **R**. **EoR** is the difference between the theoretical value **T** and actual value **E** of the system residual of the sensor. **R** is the covariance matrix of the current measurement error. This algorithm then classifies the working status of nodes into ideal, good, and poor. After this fuzzy classification, the interaction output data of the nodes are endowed with the corresponding confidence weight vector **w***_j_*(*k*). Finally, **w***_j_*(*k*) is used to complete the data defuzzification and derive the target status estimation of the network at time *k* as the output. Figure 4 illustrates the principle frame graph of FNNFS.

### 3.1. Calculation of Confidence Weight Vector

**Figure 5:**
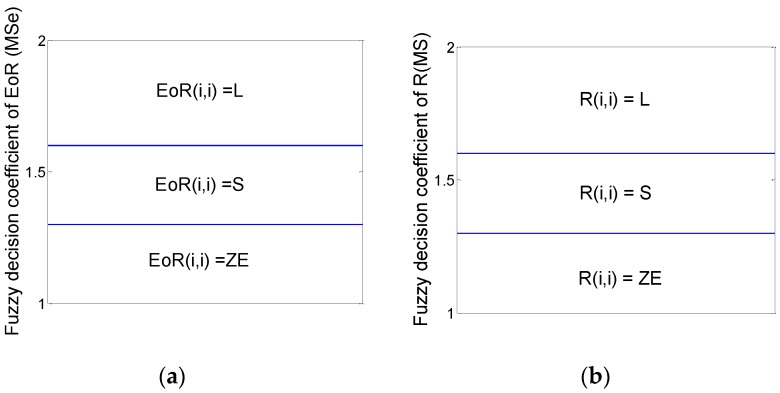
Decision functions of (**a**) **EoR** and (**b**) **R**.

The working status of node detectors in the network can be estimated using the measurement error. If the measurement error of some nodes is large or possesses a singular value (i.e., the value significantly deviates from the average value), then the data output of the node output for the current time is unreliable. A relatively low confidence weight is obtained, and the working status of the nodes is determined as poor. When the absolute value of the elements in the main diagonal of **EoR** and **R** is close to 0, the working status of the node is ideal. Otherwise, the detection error of the node is considerable, and the working status is poor. The reliability of the node data is decided. Figure 5 shows the critical functions of **EoR** and **R**.

In the figure,

(25) MSei=|EoR(i,i)mean{diag(|EoR|)}|

and

(26) $$MS_{i}=\left|R(i,i)/\mathrm{mean}(\mathrm{diag}(\left|R\right|))\right|$$

The decision relationship of **w*_j_****(k)* with **EoR***_j_(k)* and **R***_j_(k)* is shown in Table 1.

**Figure 6:**
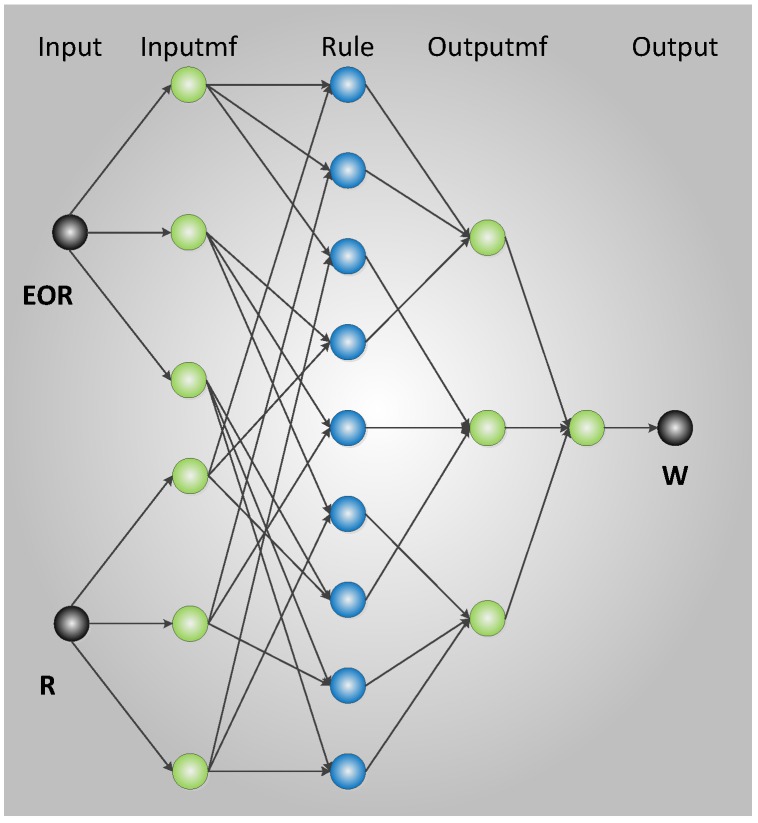
Structure of the multi-node FNN.

**w***^i^_j_*(*k*) represents the *i*-th element of the *j*-th weight vector at time *k*, and element *i* is the confidence weight of the *i*-th element in the corresponding target status vector **X**(*k*). The multi-node FNN structure is shown in Figure 6.

The following two conditions are considered to avoid possible error:

1. All the detection nodes in the network are judged by the system as having poor status at a certain time. The fusion algorithm directly adopts the node data with the lowest average of *MSe* and *MS* in Equations (25) and (26).
2. The node data are abandoned if the *MSe* of some nodes is higher than 2. If the *MSe* values of all the nodes are higher than 2, the algorithm operation stops, and an error is reported.

### 3.2. Defuzzification Data Infusion

The larger the *MSe* and *MS* values are, the poorer the status of the node will be. The defuzzification progress is implemented as follows:

(27) $${w^{i}}_{j}\left(k\right)={w^{i}}_{j}(\frac{1}{MS_{i}}+\frac{1}{MSe_{i}})$$

At time *k*, the target status output in the network is estimated to be

(28) $${\hat{X}}^{i}(k)=\arg\underset{j}{\max}({w^{i}}_{j}(k));\;\hat{X}(k)=\left[\begin{array}{c} {\hat{X}}^{1}(k)\\ {\hat{X}}^{2}(k)\\ \vdots \\ {\hat{X}}^{M}(k) \end{array}\right],$$

where $\arg\underset{j}{\max}({w^{i}}_{j}(k))$ represents the largest *i*-th element of all return target status estimation vectors at time *k*. Given that the maximum value may not be only 1 in an actual situation, the average value of the corresponding elements of all maximum values comprises the fusion estimation data.

## 4. Simulation Experiment

### 4.1. Experiment Description

In a WSN, multi-node cooperative detection is an effective means to improve network detection accuracy and system detection robustness. Networked cooperative detection consumes more resources than single-node detection. The number of detection nodes is directly proportional to the consumption of network resources. The number of detection nodes distributed in the network should be controlled within a reasonable value when tracking a single maneuvering target to achieve the best target tracking performance. This study performs calculation 100 times using the Monte Carlo method and verifies the performance of the proposed algorithm with a network of nine detection nodes.

The status estimation vector of the target is

(29) $$X={\left[\begin{array}{cccccc} x & \dot{x} & y & \dot{y} & \ddot{x} & \ddot{y} \end{array}\right]}^{T}.$$

**Figure 7:**
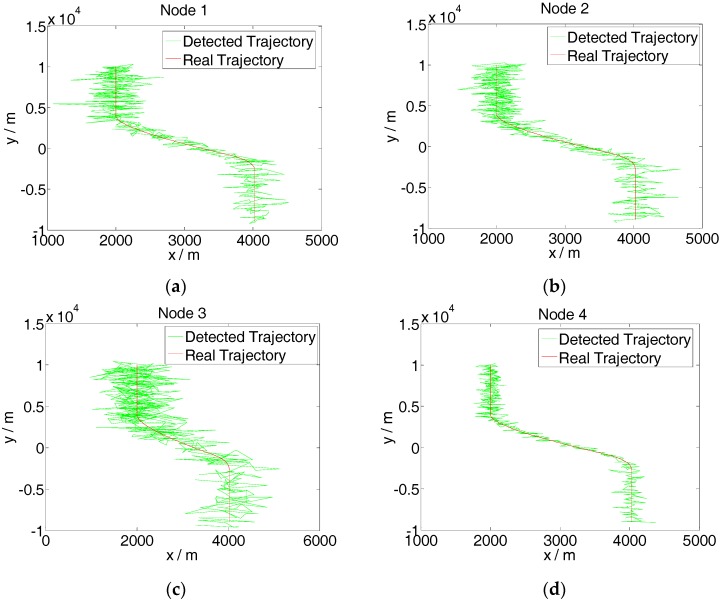
Detected target trajectory of the net nodes. (**a**) Detected trajectory of node 1; (**b**) Detected trajectory of node 2; (**c**) Detected trajectory of node 3; (**d**) Detected trajectory of node 4.

The FNN–IMM algorithm is composed of three motion models: constant velocity, acceleration, and turn. The simulation experiment is completed in a MATLAB platform. The variances of the measurement errors of nodes 1, 2, and 3 are supposed as *d*_1_^2^ = 200^2^ m^2^, *d*_2_^2^ = 200^2^ m^2^, and *d*_3_^2^ = 400^2^ m^2^, respectively, and those of the other nodes are *d_i_*^2^ = 100^2^ m^2^ (*i* = 4, 5, …, 10). Node 3 simulates a fault node, which cannot measure the coordinate of the target normally. The real motion and observation curves of the target trajectory are presented in Figure 7.

The figure shows that the target motion status consists of three uniform rectilinear motions and two accelerated motions. After the uniform rectilinear motion during the initial stage where $\dot{x}$ = 0 m/s and $\dot{y}$ = −15 m/s, the accelerated velocity after the first rotation is $\ddot{x}$ = 0.075 m/s^2^ and $\ddot{y}$ = −0.075 m/s^2^, whereas that after the second rotation is $\ddot{x}$ = −0.3 m/s^2^ and $\ddot{y}$ = −0.3 m/s^2^. The initial values are set as follows:

The Markov chain of the node:

(30) $$\Pi_{1}=\Pi_{2}=\Pi_{3}=\left[\begin{array}{ccc} 0.9 & 0.05 & 0.05\\ 0.05 & 0.9 & 0.05\\ 0.05 & 0.05 & 0.9 \end{array}\right]$$

System process noise:

(31) $$Q_{1}=Q_{2}=Q_{3}=\left[\begin{array}{ccc} 0 & 0 & 0\\ 0 & 0.001 & 0\\ 0 & 0 & 0.01114 \end{array}\right]$$

The sampling rate is

(32) $$f=\frac{1}{T}=0.5\mathrm{Hz}$$

where T represents the sampling period, and T = 2 s.

The angular velocity of the CT model is

(33) ω=0.001rad/s

The performance of the algorithm is evaluated using the location information error in the target status estimation vector, and the evaluation function is as follows:

(34) $$J=\frac{1}{mont}\sum_{i=1}^{mont}\sqrt{{\left(X(:,1)-{\hat{X}}_{i}(:,1)\right)}^{2}+{\left(X(:,3)-{\hat{X}}_{i}(:,3)\right)}^{2}}$$

where *mont* is the number of times the Monte Carlo calculation was performed, and ${\hat{X}}_{i}$ represents the estimated output vector of the network target status in the *i*-th experiment. 

### 4.2. Result Analysis

**Figure 8:**
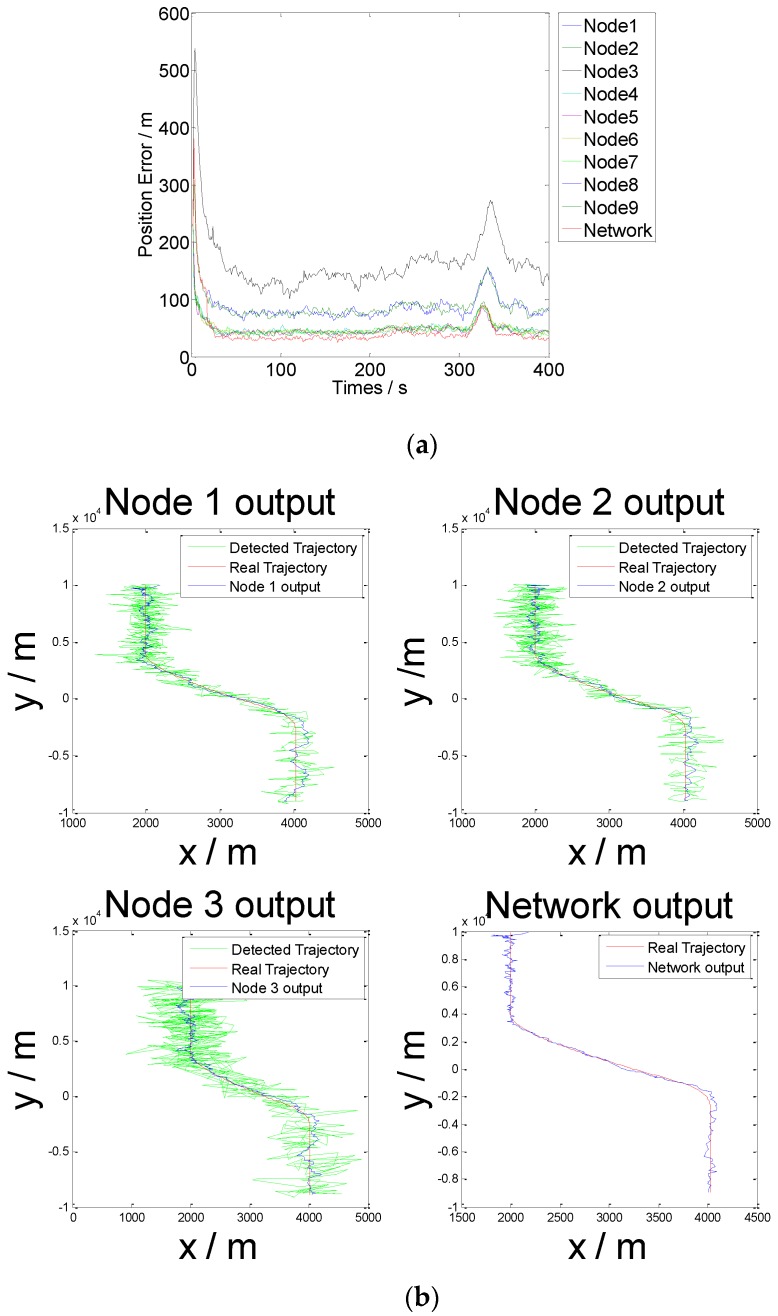
Computational results of the FNN–IMM. (**a**) Position error of each node. (**b**) Estimated trajectory of Nodes 1, 2, and 3 and the network output.

Figure 8 illustrates the algorithm operation results.

Figure 8 shows that the target position error after infusion is smaller than the position error of any node. The algorithm reduces the influence of nodes with poor working status on target tracking effects and improves the accuracy of target tracking. 

#### 4.2.1. Performance Contrast Experiment of the Single-Node FNN–IMM Algorithm

The performance of the single-node FNN–IMM algorithm is verified by comparing it with IMM-EKF, IMM-UKF, and VB-IMM [27]. We perform the calculation 100 times using the Monte Carlo method and verify the performance of the proposed algorithm with Node 3 described above. The other initial values are constant.

**Figure 9:**
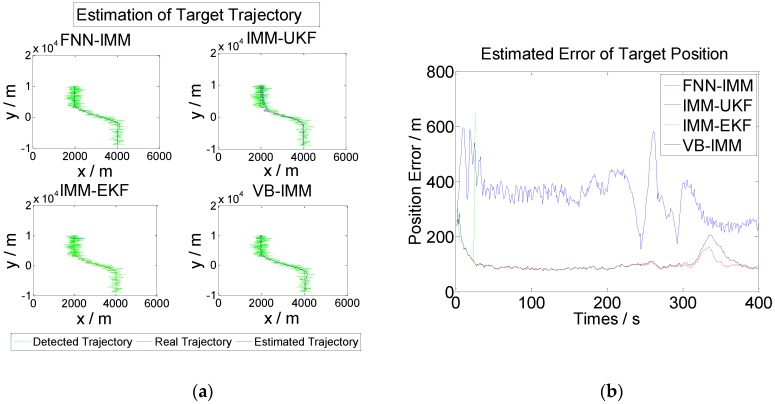
Computational results of FNN–IMM, IMM-UKF, IMM-EKF, and VB-IMM. (**a**) Mean trajectory after 100 Monte Carlo trials. (**b**) Estimation errors of target position by FNN-IMM, IMM-UKF, IMM-EKF, and VB-IMM.

Random white noise with an average value of 0 and a variance of 100 is added based on the target observation data before the beginning of each time iteration of the algorithms. In this manner, the self-adaptive capability to adjust the measurement noise of the algorithm proposed in this study can be examined. The operation results are presented in Figure 9.

Figure 9 indicates that the IMM-EKF algorithm cannot self-adaptively adjust the measurement error. The algorithm becomes ineffective and stops operating when the statistical characteristics of the measurement error change. By contrast, the proposed FNN–IMM algorithm can effectively and self-adaptively adjust the measurement error, and its applicability is higher than that of the IMM-UKF algorithm.

#### 4.2.2. Performance Verification Experiment of Multi-Node Infusion Algorithm

**Figure 10:**
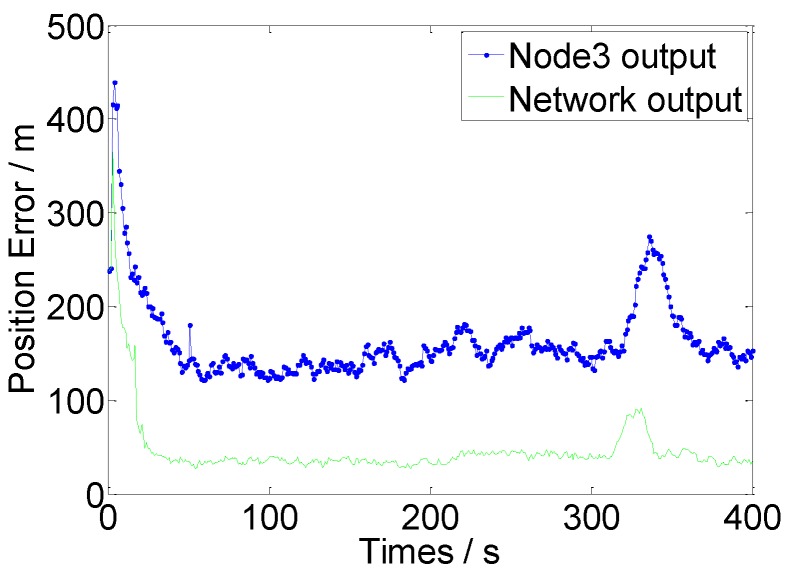
Target positional errors of the network outputs and Node 3.

The fault tolerance of the data infusion algorithm proposed in this study is verified. At the beginning of each time iteration of the algorithm, random white noise with an average value of 0 and a variance of 100 is added to the observation data of Node 3, and the other initial values are constant. In this manner, the invalidation of the node detection data is simulated. The operation results are presented in Figure 10.

Figure 10 and Table 2 demonstrate that the proposed multi-node FNN–IMM algorithm can reduce the influence of erroneous data on output results at the time of multi-node data infusion. Consequently, the fault tolerance and accuracy of the maneuvering target tracking system of the WSN are improved. 

## 5. Conclusions

An IMM multi-node target tracking algorithm based on FNN is herein proposed to solve the maneuvering target tracking problem in WSNs. This algorithm can self-adaptively adjust system measurement errors without assuming the statistical characteristics of the system measurement errors. Consequently, the proposed algorithm can effectively overcome the shortcoming of the IMM algorithm of requiring a priori knowledge of the system measurement errors. The information infusion system of the FNN algorithm reduces the influence of poor nodes on target status estimation during the multi-node infusion stage. The fault tolerance mechanism is proposed according to the possible faults in data infusion, thus improving the fault tolerance of the algorithm. The simulation experiment indicates that the multi-node FNN–IMM algorithm exhibits high practicability.

## Figures and Tables

**Table 1:** Decision table of **w***_j_*(*k*).

	R*_j_*(*i,i*)	ZE	S	L
EoR*_j_*(*i,i*)				
ZE		**w***^i^_j_*(*k*) = 1	**w***^i^_j_*(*k*) = 1	**w***^i^_j_*(*k*) = 0.5
S		**w***^i^_j_*(*k*) = 1	**w***^i^_j_*(*k*) = 0.5	**w***^i^_j_*(*k*) = 0
L		**w***^i^_j_*(*k*) = 0.5	**w***^i^_j_*(*k*) = 0	**w***^i^_j_*(*k*) = 0

**Table 2:** Position accuracy of the multi-node FNN–IMM and the single-node FNN–IMM.

	Multi-Node Output of FNN–IMM	Single Node Output of FNN–IMM
Average Position Error ±Δ(x,y)	46.9680	162.6039
Average Error of *x* axis ±Δx	34.6952	119.7772
Average Error of *y* axis ±Δy	34.7728	120.1503
